# Not guilty by reason of insanity: characteristics of psychotic murders and murderers

**DOI:** 10.1192/j.eurpsy.2025.1527

**Published:** 2025-08-26

**Authors:** A. Yaron Antar

**Affiliations:** 1 Criminology, The Max Stern Yezreel Valley College, Yezreel Valley, Israel

## Abstract

**Introduction:**

Some acts of murder are committed out of insanity by individuals suffering from psychotic mental disorders. According to the law in many countries, these individuals do not have criminal responsibility. They are defined as not guilty due to insanity and therefore cannot be punished.

**Objectives:**

This study aims to explore the sociodemographic, psychiatric, criminal and forensic characteristics/factors of insanity murders and murderers. This examination has not yet been conducted in Israel, which is a multicultural country with social, religious, ethnic complexity.

**Methods:**

This study examined the hospital records (investigation material, indictments, admission summaries, and expert testimonies) of all 80 inpatients who had committed murder and been hospitalized in the maximum security unit from its opening in 1997 until 2021, and were found not guilty due to insanity.

**Results:**

***Demographics characteristics*** (at the time of the offense). The participants were men ages 18-85 (M=36.11, SD=11.84). 58.8% were born in Israel, 21.3% immigrated from the former Soviet Union, 6.3% from Ethiopia, and 13.8% from other countries. The majority (82.5%) were Jewish, 16.4% were Arab. Most lived in urban centers (86.3%). Most were not married at the time of the offense: Single (62.5%), divorced (21.3%). Only a minority was married (16%).

***Psychiatric characteristics.*** Most participants were diagnosed with schizophrenia (90%); had at least one hospitalization prior to the offense (70%), and had a previous hospitalization due to violence. Most of them were not compliant with psychiatric treatment and follow-up (only one participant was fully compliant). In most cases (74%) pre-murder deterioration was recorded.

***Criminal characteristics.*** At least 52.5% had a criminal record prior to committing the offense (a higher number is possible, the information is based on the medical file only, there was no access to an official criminal record).

***Murder/forensic characteristics.*** At least 48.8% there was no prior planning. In most cases (66.6%) the motive described was paranoid delusions and only in 5% there is command hallucinations. In most cases the murders took place in the home of the victim and/or the assailant (67.5%), one person was murdered (91%) and the victim was known to the assailant (88.8%), most of them a family member (61.3%). In at least 75% of the cases there was a brutal murder with multiple stabbings, use of multiple means/actions, abuse of the body or dismemberment. Following the murder, 58.3% of the assailants remained at the site of the crime and/or called for help, 27.4% left the site (no information for the remaining subjects).

**Conclusions:**

The findings are consistent with existing knowledge and may assist in identifying at-risk populations, develop and implement relevant prevention programs as well as improving the therapeutic continuum from hospitalization to the community.

**Disclosure of Interest:**

None Declared

